# Identity, Structure and Compositional Analysis of Aluminum Phosphate Adsorbed Pediatric Quadrivalent and Pentavalent Vaccines

**DOI:** 10.1016/j.csbj.2018.11.006

**Published:** 2018-11-29

**Authors:** Kristen Kalbfleisch, Sasmit Deshmukh, Carmen Mei, Moriam Ore, Wayne Williams, Ibrahim Durowoju, Jessica Duprez, Sylvie Morin, Bruce Carpick, Marina Kirkitadze

**Affiliations:** a Analytical Sciences, Sanofi Pasteur Canada, 1755 Steeles Avenue West, Toronto, Ontario, Canada; b Department of Chemistry, York University, 4700 Keele Street, Toronto, Ontario, Canada,; c SGS Canada, Biopharmaceutical Services, 6490 Vipond Drive, Mississauga, Ontario, Canada

**Keywords:** Adsorbed vaccines, Identity, Protein conformation, Particle sizing, FTIR, SEM, Fluorescence

## Abstract

**Purpose:**

The goal of this study is to set an empirical baseline to map the structure-function relation of the antigens from the commercialized vaccine products.

**Methods:**

To study the structural changes of protein antigens after adsorption several analytical tools including DLS, FTIR, Fluorescence, LD, and SEM have been used.

**Results:**

All antigens have shown wide range of hydrodynamic diameter from 7 nm to 182 nm. Upon adjuvantation, the size distribution has become narrow, ranging from 10 to 12 μm, and has been driven by the derived diameter of aluminum phosphate (AlPO_4_) adjuvant. Further to examine size and morphology of adsorbed antigens, SEM has been used. The SEM results have demonstrated that the AlPO_4_ adjuvant suspension and adsorbed proteins consist of submicron particles that form a continuous porous surface. Diphtheria Toxoid (DT), Tetanus Toxoid (TT), and chemically-modified Filamentous Haemagglutinin (FHA) have shown surface adsorption to AlPO_4._ Secondary structure alpha-helix and beta-sheet content of DT and TT has increased after adsorption to AlPO_4_ adjuvant as shown by FTIR, whereas no significant changes were noted for other protein antigens. The results from Intrinsic Fluorescence have shown a structural rearrangement in DT and TT, consistent with the FTIR results. Multivalent vaccine product identity has been determined by FTIR as unique fingerprint spectrum.

**Conclusion:**

The globular proteins such as DT and TT have shown changes in secondary structure upon adsorption to AlPO_4_, whereas fibrillar protein FHA has not been affected by adsorption. FTIR can be used as a lean technique to confirm product identity at different manufacturing sites.

## Graphical Abstract

**Figure:**
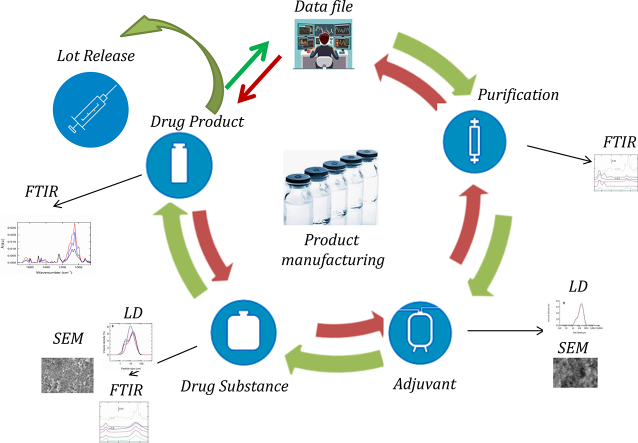

## 1 Introduction

Traditionally, complex biological products such as vaccines presented unique challenges to implementation of even rudimentary characterization packages; thus, the product was defined almost exclusively by its manufacturing process, i.e.,if the process remains unchained, the product should be the same. The advances in technology allowed the application of more comprehensive characterization packages for products such as adsorbed combination vaccines, containing several antigens in a single formulation to protect against more than one disease. The application of extensive characterization packages can now extend beyond simply characterizing the purified proteins, to include product intermediates [1,2], and adsorbed protein drug substances and adjuvanted vaccine formulations [[3], [4], [5], [6]]. As discussed previously [7] characterization of vaccine attributes at both the drug substance and drug product stages have progressively higher criticality with respect to product supply, safety andimmunogenicity. For vaccines, this encompasses not only protein antigens, but also adjuvants, and adjuvanted and multivalent product formulations. Factors that can affect safety and efficacy critical quality attributes and critical material attributes may include, but are not limited to, protein adsorption and conformation, size distribution and morphology of adsorbed drug substances (DS). Presented here, to assess these attributes, are several of analytical tools with the capability of characterizing multivalent vaccines and their components, as well as lot-to-lot consistency. The principle applied here is that the quality of subsequent batches is the consequence of the strict application of a quality system and of a consistent production of batches, which can be demonstrated using state-of-the-art and non-animal methods [8].

Many reports in the literature demonstrate that protein adsorption to an adjuvant can alter its conformation [4,6,9,10], and either stabilize [11], destabilize [3,9,12], or show no effect [5] on conformation. This highlights the importance of analytical tools capable of monitoring these possible changes in protein antigens throughout the manufacturing process.

Multivalent vaccines offer better protection against certain diseases such as pertussis [13], and manufacturing of combination products with the same immunogenicity and safety profile as each of its individual component vaccines is a considerable challenge [14]. Development of combination vaccines requires a careful assessment and selection of adjuvant(s) and process steps including formulation of the intermediates and final product. Furthermore, there may be physicochemical or immunological interference between any or all of the components [15].

The goal of this study is to set an empirical baseline to map the structure-function relation of the antigens from the vaccine products. For that purpose the samples analyzed here were the commercialized vaccines proven to be immunogenic in clinic. Hence this study was designed to understand the differences between the pre-adsorbed and adsorbed antigens used to formulate vaccine product. This biophysical toolset was not explored in the past for the vaccine components produced at manufacturing scale. This will serve a as a basis to understand any future changes in the manufacturing process, facility, or site.

To provide a more comprehensive analysis of current manufacturing processes, samples of intermediate pre-adsorbed protein antigens, adsorbed drug substances, and drug products were examined using a panel of methods. These included dynamic light scattering (DLS), laser diffraction (LD), scanning electron microscopy (SEM), Fourier transform infrared (FTIR) spectroscopy, and intrinsic fluorescence (IF) spectroscopy. These non-routine characterization tests were applied for the purpose of product knowledge.

Particle size can be an indication of both process consistency and product stability, and can be a quality attribute used in the characterization of vaccine and vaccine components [16]. DLS was utilized to characterize the size of pre-adsorbed protein antigens, while LD was applied to particle sizing of adjuvant and adjuvanted dug substances.

As antigen protein conformation may affect the presentation of epitopes, the effect of adjuvantation on protein higher order structure was analyzed. FTIR was utilized to measure secondary structure content, and IF to examine tertiary structure conformation.

With the objective of a comprehensive characterization of multivalent vaccines and their components, a novel SEM approach to the visualization of adjuvant size and morphology was developed. Use of low vacuum SEM imaging mode allows characterization of non-conductive biomaterials [15]. This allowed an investigation of the effect of adsorbed proteins on adjuvant morphology and packing density of the suspension that in turn could be used to gain product knowledge and characterize adjuvantation step of the manufacturing process.

Finally, although various multivalent vaccines may contain similar antigen profiles, minor variations in their composition or formulation may be detected by a sufficiently sensitive and selective method. FTIR could be used to measure a signature spectrum, not only for individual adsorbed monovalent drug substances, but also for several multivalent vaccine drug products. Thus, FTIR can be used to identify very similar drug products.

## 2 Materials and Methods

### 2.1 Reagents and Materials

All samples examined in this study were manufactured in-house, including adjuvant, pre-adsorbed and adsorbed protein samples (i.e. drug substances), and final vaccine products. All protein antigens were purified from the respective pathogen. The proteins in solution, either purified or chemically-modified were hereafter referred as pre-adsorbed antigens, which denotes a specific manufacturing step. Upon formulation with aluminum phosphate adjuvant (AlPO_4_), the proteins were referred as adsorbed antigens of drug substances.

Quadracel™ contains Diphtheria Toxoid (DT), Tetanus Toxoid (TT), acellular pertussis proteins: Pertussis Toxoid (PT), Filamentous Haemagglutinin (FHA), Pertactin (PRN), Fimbriae types 2 and 3 (FIM), inactivated poliomyelitis vaccine (IPV) type 1 (Mahoney), type 2 (MEF-1) and type 3 (Saukett) as active ingredients [17]. The pI values of these antigens measured by Capillary Electrofocusing Imaging are summarized in Table S1. In addition to the above ingredients, Pentacel [18] and Pediacel [19] also contain purified polyribose ribitol phosphate capsular polysaccharide (PRP) of Haemophilus influenzae type b covalently bound to TT (used as carrier protein). The previously listed toxoids are a form of respective toxins chemically-modified with formaldehyde. Additionally, these vaccines include AlPO_4_, 2-phenoxyethanol, and polysorbate 80 [[17], [18], [19]]. All pre-adsorbed antigens were in phosphate buffer, except pre-adsorbed DT, which was in 0.9% saline. The molar ratio of each protein antigen is listed in Table S2.

### 2.2 Dynamic Light Scattering (DLS)

All DLS measurements of particle size distribution of pre-adsorbed antigens were performed using a Nanotrac 150 instrument (Microtrac, Montgomeryville, PA, USA). All the samples were measured at room temperature at 20 fold dilution using MilliQ water, hence viscosity of water was used for the data analysis. Total volume for all measurements was 600 μL. Nanorange mode was enabled for appropriate analysis of the particle sizes below 20 nm. The data acquisition and analysis were done by Microtrac Flex software. The particle size was reported as hydrodynamic diameter in nm, with 1 decimal point. Coefficient of variation for the qualified generic DLS method was ranging from 5 to 10% for DT, TT, FHA, FIM, and 15% and above for PRN and PT.

### 2.3 Laser Diffraction (LD)

All measurements of particle size distribution of adjuvant, adsorbed antigens and multivalent vaccine products were performed using a Mastersizer 3000 instrument (Malvern Instruments Ltd., Westborough, MA, USA), operating in a dynamic range of 0.01 to 3500.00 μm. Particle size distributions in solutions and suspensions were quantitatively determined by measuring the angular variation in intensity of light scattered from a laser beam passing through a dispersed particulate sample. The reportable value is Derived Diameter (Dv), which is the particle size (in μm) for a specific percentile of the cumulative size distribution. Particles were measured at room temperature using the built-in “non-spherical” option within the software, and the average Dv10, Dv50 and Dv90 values of 5 measurements were reported in μm with 1 decimal point. The coefficient of variation for the qualified LD assay was in the range of 5–7% for the adsorbed antigens. Data re-plotting was performed using SigmaPlot.

### 2.4 Fourier Transform Infrared (FTIR) Spectroscopy

FTIR spectroscopy was performed using a Vertex 70 FTIR Spectrometer (Bruker Optics, Bremen, Germany), equipped with a cryogenically-cooled MCT (mercury-cadmium-telluride) detector and a BioATRII sampling accessory. A sample volume of 20 μL was loaded onto the sample cell and the spectra were collected at a resolution of 0.4 cm-1 at 25 °C with a wavenumber accuracy of 0.01 cm^−1^ at 2000 cm^−1^. The samples were allowed to stabilize for 1 min on the ATR crystal. Background (Milli-Q water) and sample measurements were conducted with each reported measurement representing an average of 200 scans. Data acquisition and analysis were performed using the OPUS 6.5 software (Bruker Optics, Bremen, Germany). OPUS automatically subtracts the background signal from the sample to produce the spectrum for the analyte. All measurements were carried out at 25 °C using a Haake DC30/K20 temperature controller (Karlsruhe, Germany). After acquiring the FTIR spectra, the baseline was corrected by removing the scattering signal using the OPUS software. Quant2 software (Bruker Optics) was used to estimate secondary structure with an error of 5.5% for alpha-helix content and 4.4% for beta-sheet content. The second derivative spectrum was generated using the Savitzky-Golay algorithm, which allowed simultaneous smoothing of the spectrum. Arithmetic manipulations and re-plotting were performed using SigmaPlot.

### 2.5 Scanning Electron Microscopy (SEM)

SEM was used to examine the morphology and size of adjuvanted protein antigens. All measurements were performed using FEI Quanta 3D SEM (ThermoFisher Scientific, Waltham, MA, USA) in the Imaging Facility at York University. Low Vacuum SEM mode was used to image the dried adjuvanted samples and was accomplished by centrifugation of the sample at 6000 rpm, followed by removal of the supernatant. Sodium chloride, a residual of adjuvant production process, may interfere with SEM characterization of the microstructure, and therefore, samples were rinsed prior to analysis. Pellets were then rinsed 3 times with LC-grade water. The rinsed pellets were then immobilized by smearing a 10 μL aliquot of the adjuvant suspension on a glass microscope slide. The samples were imaged using a low vacuum secondary electron detector.

### 2.6 Intrinsic Fluorescence Spectroscopy (IF)

IF spectroscopy was performed using Varian Cary Eclipse spectrophotometer (Agilent Technologies, Santa Clara, CA, USA). Intrinsic fluorescence, a dye-free method to evaluate changes in aromatic amino acid residues (fluorophores) within proteins, was used to probe changes in the local environment as a result of adsorption onto the surface of AlPO_4_ adjuvant. All protein samples were excited at 285 nm and emission spectra were recorded in 300 to 400 nm region using multi-cell holder accessory of Cary eclipse. Measuring parameters such as slit width were optimized for each sample to obtain maximum fluorescence intensity.

## 3 Results

### 3.1 Particle Size, Morphology, and Composition

**Fig. 1:**
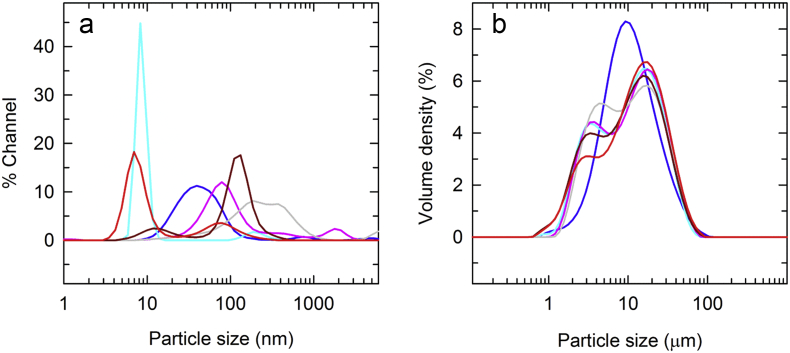
Particle size distribution of (a) pre-adsorbed and (b) adsorbed protein antigens by DLS and LD respectively. Representative traces include PRN (cyan), DT (red), FHA (blue), FIM (pink), TT (brown), and PT (grey) antigens. (For interpretation of the references to colour in this figure legend, the reader is referred to the web version of this article.)

Size distribution profiles of pre-adsorbed and adsorbed proteins were measured using DLS and LD respectively (Fig. 1). DLS was used for pre-adsorbed samples while LD was used for adjuvants and adsorbed vaccines (Fig. 1 and S1). The size distribution profiles as determined by DLS for each of the pre-adsorbed antigens are shown in Fig. 1. These antigens will ultimately be formulated into a multivalent vaccine with protection against Pertussis, Diphtheria and Tetanus. On an average, particle sizes ranged from 10 to 200 nm, and show the diversity of size from antigen to antigen. The polydispersity index for each antigen is listed in Table S3. Each showed a unique distribution profile (see Table S3 for hydrodynamic diameter values).

AlPO_4_ was used as an adjuvant in the adsorbed form of the antigen proteins, and its particle size (Dv50 value) was in the range 9–13 μm (Fig. S1a).

The size distribution profiles depicted in Fig. 1b were representative of the three lots of each product analyzed. With the exception of adsorbed FHA, each monovalent adsorbed antigen profile showed two major peaks and a broad size distribution ranging from approximately 1-100 μm. By contrast, adsorbed FHA showed one major peak and a narrower size distribution. Final drug product (data not shown) that includes all six adsorbed protein antigens was similar in size distribution to most of the monovalent adsorbed antigens.

**Fig. 2:**
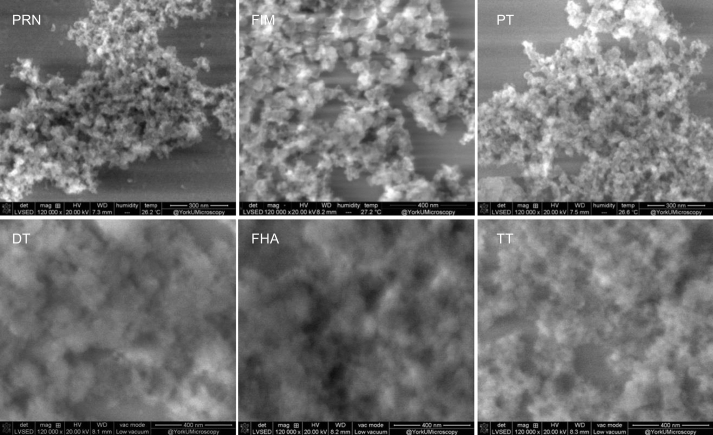
Low vacuum SEM images of AlPO4 adsorbed antigens.

In addition to particle size, the previously unexplored characteristic of particle morphology was visualized for the first time for an AlPO_4_ adjuvant suspension, as well as for each of the adsorbed monovalent antigens. Panel b in Fig. S1 depicts a low vacuum SEM image of AlPO_4_ adjuvant, most prominently highlighting the formation of irregularly shaped agglomerates comprised of smaller particles. In Fig. 2, six AlPO_4_ adsorbed monovalent antigens are compared using low vacuum SEM, in which finer features of the surface can be visualized [20]. The SEM images of DT, TT, and FHA were not sharp compared to the other three antigens although they were recorded under the same conditions. This can be assigned to lower conductivity surfaces. The changes in conductivity observed for DT, TT, and FHA samples indicated that the adsorbed layer onto the surface of AlPO_4_ was likely more dense. The SEM images shown here were representative of the whole sample, see Fig. S1c for additional SEM images.

As shown in Fig. 2, adsorbed protein samples and AlPO_4_ adjuvant were similar in morphology when imaged in low vacuum mode, and no changes to adjuvant were observed after antigen adsorption.

### 3.2 Secondary Structure

**Fig. 3:**
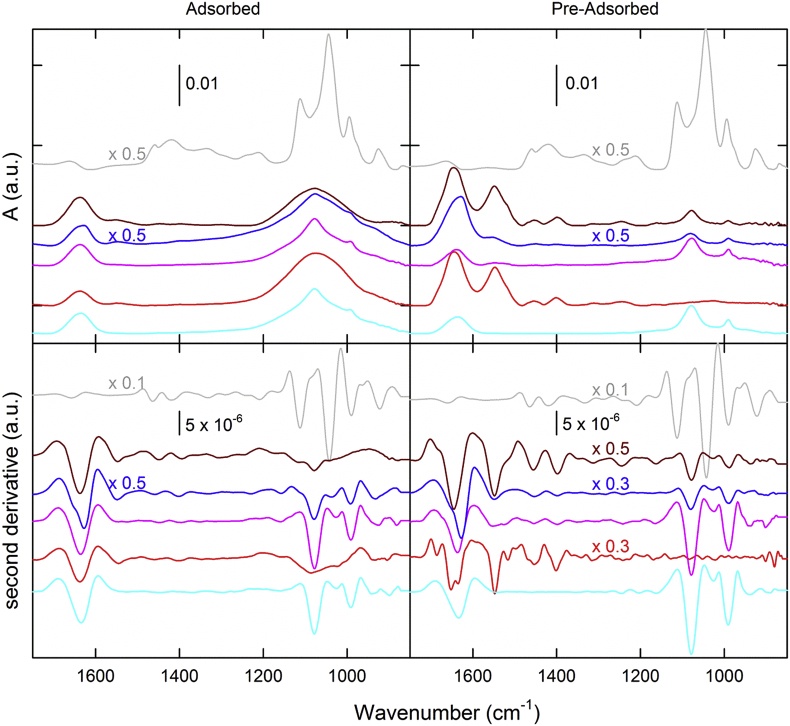
Representative FTIR spectra (top panels) and their calculated second derivatives (bottom panels) for all adsorbed (left panels) and pre-adsorbed (right panels) antigens. Some of measured spectra and calculated first derivative spectra are rescaled due to high signal intensity. Representative traces include PRN (cyan), DT (red), FHA (blue), FIM (pink), TT (brown), and PT (grey) antigens. (For interpretation of the references to colour in this figure legend, the reader is referred to the web version of this article.)

As previously discussed, proteins adsorbed on the surface of adjuvant particles may undergo conformational changes. Higher order structural changes following AlPO_4_ adsorption were characterized by different spectroscopic methods: FTIR (secondary structure), and IF (tertiary structure). FTIR spectroscopy was used to probe the conformational changes associated with adsorption by monitoring shifts in secondary structure from pre-adsorbed to adsorbed in purified monovalent antigens (Fig. 3). Table S4 indicates the changes in alpha helix and beta sheet content upon adsorption.

An increase in both alpha-helix and beta-sheet content were observed for DT and TT upon adsorption to AlPO_4_ (Table S4). However, for PRN, FIM and FHA the changes detected were within the experimental error and hence deemed insignificant. While pre-adsorbed and adsorbed PT antigen did not show any spectral change (Fig. 3). FTIR spectra for all six adsorbed monovalent antigens are presented, and supplemented with the second derivative spectra to highlight regions of variability. All protein antigens characterized, except PT, also showed a broad peak around 1078 cm^−1^ for AlPO_4_ adjuvant in the adsorbed form. Some small changes were also observed in protein backbone and sidechain around 1400 and 1453 cm^−1^ as a result of adjuvantation.

**Fig. 4:**
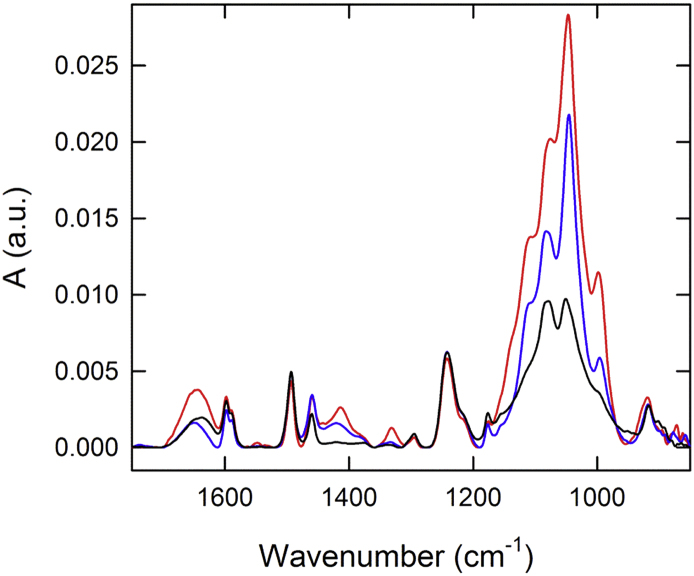
Overlay of FTIR spectra for Pediacel® (red trace), Pentacel® (blue trace) and Quadracel™ (black trace). (For interpretation of the references to colour in this figure legend, the reader is referred to the web version of this article.)

All drug substances, as well as the final multivalent product samples (Fig. 3, Fig. 4) showed unique spectral features. As shown in the upper left panel in Fig. 3, similar spectral features are observed in FHA, FIM and PRN, whereas by contrast, DT and TT were similar to some extent. In cases where unambiguous distinction is difficult by comparing spectra, calculated 2nd derivative spectra can elaborate additional spectral information, as shown in the lower panel of Fig. 3. In this analysis, the differences emerge within the amide I and II regions for each of the tested drug substances in the pre-adsorbed versus adsorbed forms. This region highlights the changes in β-sheet, turns and α-helices at approximately 1624, 1676 and 1654 cm-1, respectively. Secondary structural content may be influenced by adsorption to AlPO_4_ as a result of changes to the local environment of the antigens, which can also be detected by the shifts in the FTIR peak positions. The low frequency region (between approximately 1076 and 990 cm-1) consists mainly of contributions from adjuvant and phosphate buffer.

### 3.3 Identity

The combination or multivalent vaccine products Quadracel™, Pentacel®, and Pediacel® [[18], [19], [20]] all contain AlPO_4_ as an adjuvant and have many antigens in common. As a result, the spectral features of these combination products are quite similar (Fig. 4), yet small but detectable differences were observed. For instance, the peak representative of the P-O stretch (around 1079 cm^−1^) had higher absorbance in Quadracel™ when compared to Pentacel® or Pediacel®, the latter showing a shift in this peak (to 1083 cm^−1^). Another spectral difference was observed at 1420 cm^−1^ in Pentacel® and Quadracel™, where both showed a broad shallow peak in contrast to the sharper peak detected at 1414 cm^−1^ in Pediacel®.

### 3.4 Tertiary Structure

**Fig. 5:**
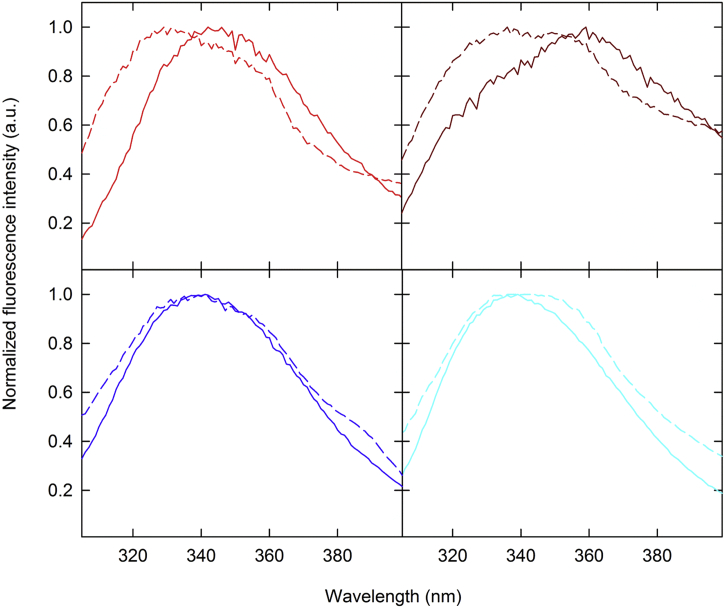
Intrinsic fluorescence emission spectra of DT (red), TT (brown), FHA (blue), and PRN (cyan) antigens in pre-adsorbed (solid traces) and adsorbed (short dashed traces) forms. (For interpretation of the references to colour in this figure legend, the reader is referred to the web version of this article.)

Intrinsic fluorescence spectroscopy (IF) was used to probe effect of adsorption on tertiary structure of the proteins. IF emission spectra of DT and TT revealed that adsorbed form of the protein has hypsochromic shift in tryptophan fluorescence emission as compared to pre-adsorbed antigens (Fig. 5).

FHA and PRN showed no significant shift whereas FIM and PT did not show fluorescence emission signal in either form.

## 4 Discussion

Vaccines are complex formulations containing multiple components such as protein antigens, adjuvants, excipients, stabilizers etc., and hence they form complex interations within the matrix. Therefore it is imperative to perform identity, compositional and structural analysis as a mean of quality control as well as to gain product knowledge. This study focuses on these aspects of the vaccine components and products through set of biophysical methods. As per ICH Q6B, it is important to understand and characterize physico-chemical properties of protein antigens such as higher order structure, purity, identity, biological activity, post-translational modifications [21].

The results from particle size distribution suggest that AlPO_4_ adjuvant primarily affects the overall size of the adsorbed protein antigens (Fig. 1 and S1a). It also appears that the majority of particle size distribution profiles of the adsorbed protein antigens have some variability from particle size of AlPO_4_ as a result of adjuvantation (Fig. 1b and S1, and Table S3). Apparently, particle size is important in the uptake of particles by antigen presenting cells [16], and the size of 10 μm is optimal [16]. This is in agreement with the particle sizes (Dv50 values) of adsorbed protein antigens found in this study (Table S3). The adjuvant suspension consists of small submicron particles that form continuous porous surfaces, and dense surface texture, which may impact antigen adsorption (Fig. S1b) and therefore there is some variability for particle size distribution of adsorbed protein antigens.

SEM images demonstrate that AlPO_4_ suspension and adsorbed proteins consist of small submicron particles that form a continuous porous surface (Fig. 3 and S1b). The approximate overall size of these particles is of ~4–5 μm as measured by SEM (Fig. S1c) and ~8–14 μm as shown by LD (Fig. 1). These differences were due to experimental conditions, such as hydration level of the adjuvant suspension and the presence of a vacuum for SEM measurements. As shown internally by Electrochemiluminescence and ELISA assays (data not shown), DT, TT, and FHA show high % adsorption (about 90%) to AlPO_4_, whereas % adsorption of PRN, PT, and FIM is low (below 30%). Adjuvant particles with DT, TT, and FHA appear larger in size possibly denser due to interaction between proteins and adjuvant surface. The adjuvant appears to be coated with the protein (DT, TT, or FHA) making the surface less conductive, and resulting in less sharp images.

FTIR spectroscopy was used to probe secondary structural changes as a result of adjuvantation due to its ability to measure adjuvanted samples using ATR crystal. In FTIR spectra, individual peaks represent vibrational modes of the molecules under study and the alteration in the local environment of these molecules is detected by shifts in the peaks or the appearance or disappearance of certain peaks. This information was used while acquiring and analyzing spectra of these vaccine components. Drug substances that primarily consist of single antigens can be characterized using FTIR before and after adjuvant formulation. All of the vaccine antigens tested are purified proteins and thus share some fundamental FTIR spectral features. Moreover, the degree of adsorption to AlPO_4_ adjuvant may differ among antigens due to concentration, pI, or other factors and this may complicate the analysis of spectral features in adsorbed samples. Besides PT all other protein antigens showed spectral changes from pre-adsorbed to adsorbed formulations (Fig. 3). This suggests that PT likely does not adsorb to AlPO_4_ surface which is also in agreement with SEM data. Besides PT and PRN all other protein antigens in the pre-adsorbed form have amide II signal, which disappears due to adjuvantation, this indicates structural changes involving amide II region. The toxoids, DT, TT, and FHA were all adsorbed to the surface of AlPO_4_, however, only DT and TT showed an increase in secondary structure content (Table S4), consistent the findings reported in literature [11] for the effect of adjuvant on protein structure. This is most likely due a difference in overall structure of the antigens, such as globular structure of DT and TT, versus elongated fibrillar structure of FHA. Secondary structure elements of DT, TT, FHA, PRN, PT, and FIM detected by FTIR were consistent with the structure of PRN [22], Diphtheria Toxin [23], Tetanus Toxin [24], Pertussis Toxin [25], and with the models of FHA [26,27] and FIM [27] reported in literature. In addition, the adsorption of DT and TT to AlPO_4_ induced additional rearrangement due to surface interaction. Whereas, for FHA, it appears that adsorption does not facilitate additional structural rearrangement. PT, consisting of globular domains that are chemically-modified, did not exhibit any changes similar to DT and TT. Therefore the detoxification alone does not explain differences in protein secondary structure upon adsorption.

The results obtained by FTIR for DT, TT, and FHA are in agreement with changes observed in the intrinsic fluorescence emission spectra for these proteins. As shown in Fig. 5, a band broadening observed for the adsorbed antigens likely occurred due to altered solvent interactions with each fluorophore (Fig. 5). The overall hypsochromic shift in adsorbed DT and TT indicate that tryptophan residues are more buried and have less solvent access, which could indicate these proteins are more folded than their pre-adsorbed forms or these residues are shielded by the adjuvant surface. For PT, PRN, and FIM, the presence of AlPO_4_ did not induce any significant changes as shown by FTIR and intrinsic fluorescence.

A forced degradation study showed that a decrease in antigenicity of adsorbed TT using chemiluminescence (MSD) was consistent with a decrease of thermal transition temperature measured by DSC for pre-adsorbed (Fig. S2) and by nanoDSF for adsorbed TT (Fig. S3). As such, for the adsorbed TT stored at 45 °C the antigenicity decreased from 4586 μg/mL at time zero to 2600 μg/ml at 17 weeks, whereas, at 55 °C the antigenicity decreased to 0.037 μg/ml in just 1 week.

As demonstrated in a recent study [28], the FTIR spectra of both adsorbed monovalent antigens and multivalent vaccine products (Fig. 3 and Fig. 4) showed rich information that can be recorded as a spectral fingerprint for each tested sample allowing FTIR spectroscopy to be used as a lean technique to verify the bulk drug substance identity prior to formulation, and in-process test to verify vaccine product identity prior to filling. Although multivalent vaccines can appear to be very similar in formulation, the addition of *Haemophilus influenzae* conjugate component and excipients in the formulation results in a unique signature profile for each product tested thus far.

To summarize, FTIR can be used as lean technique to verify identity of the bulk drug substance prior to formulation and also to gain knowledge about changes to protein antigens as a result of adsorption.

The findings presented here will be used for future comparability studies to assess the effects of process optimization, changes in manufacturing facilities and sites [29].

## 5 Conclusions

In this study, a toolset of biophysical techniques were applied to the analysis of pre-adsorbed and adsorbed vaccine antigens, drug substances, and drug products so as to set an empirical baseline to map the structure-function relation of the antigens from the commercial vaccine products. As shown by SEM, the AlPO_4_ adjuvant suspension consists of small submicron particles that form a continuous porous surface. As shown by FTIR, secondary structure alpha-helix and beta-sheet content of DT and TT increased after adsorption to AlPO_4_ adjuvant, whereas no significant changes were noted for other protein antigens besides structural changes within the amide region. Similarly, SEM showed strong interactions between AlPO_4_ adjuvant and DT, TT, and FHA. Finally, FTIR spectroscopy can be used as a direct method capable of identifying final drug product without desorption using a unique spectrum (fingerprint) generated by a combination of protein antigens and excipients.

## Conflicts of Interest

The authors declare no conflict of interest. Kristen Kalbfleisch, Sasmit Deshmukh, Wayne Williams, Ibrahim Durowoju, Jessica Duprez, Carmen Mei, Bruce Carpick, and Marina Kirkitadze are employees of Sanofi Pasteur, and Sylvie Morin and Moriam Ore are the employees of York University and have no other relevant affiliations or financial involvement with any organization or entity with a financial interest in or financial conflict with the subject matter or materials discussed in the manuscript. Thus includes employment, consultancies, stock ownership or options, or royalties.

No writing assistance was utilized in the production of this manuscript.

## Appendix A Supplementary data

Supplementary material Image 1
