# Supplementary material for: Identity, Structure and Compositional Analysis of Aluminum Phosphate Adsorbed Pediatric Quadrivalent and Pentavalent Vaccines
Source: Comput Struct Biotechnol J. 2018 Nov 29;17:14–20. doi: 10.1016/j.csbj.2018.11.006 (PMC6297905; doi:10.1016/j.csbj.2018.11.006)
Supplement: Supplementary file 1 — Supplementary material [file mmc1.docx]

**Supplement**

**Identity, Structure and Compositional Analysis of Aluminum Phosphate Adsorbed Pediatric Quadrivalent and Pentavalent vaccines**

Kristen Kalbfleisch, Sasmit Deshmukh, Carmen Mei, Moriam Ore, Wayne Williams, Ibrahim Durowoju, Jessica Duprez, Sylvie Morin, Bruce Carpick, and Marina Kirkitadze

a

b

c


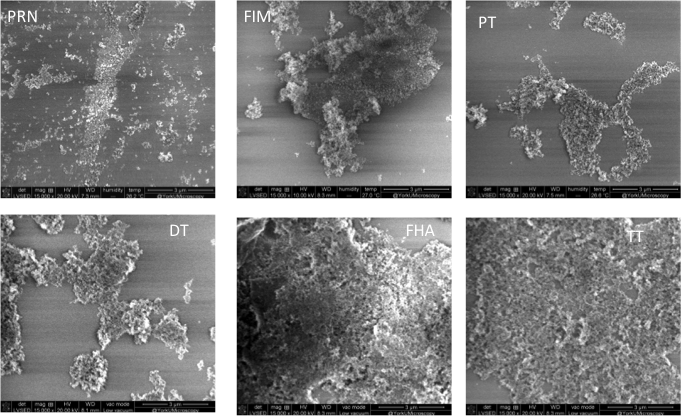


**Figure S1:** LD size distribution profile (a) and SEM morphology AlPO_4_ adjuvant (b) and adsorbed drug substances at low vacuum (c)

**Figure S2**: DSC thermogram of pre-adsorbed TT stability timepoints at time zero, 2 weeks at different incubation temperatures, 45°C and 55°C. Thermal transition temperature and enthalpy of TT decreased at elevated incubation temperatures as shown above. The black trace is measured thermogram, whereas red trace is a non-two state fitting.


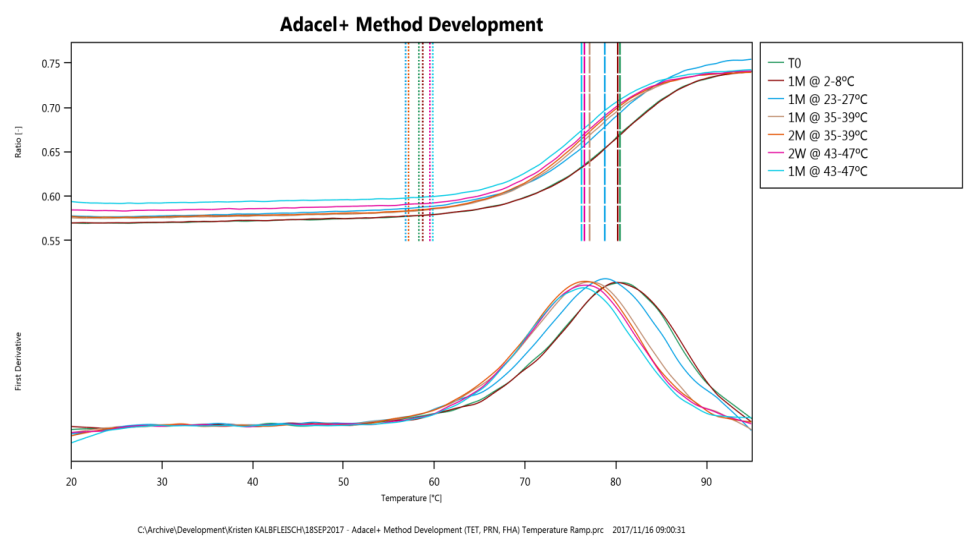


**Figure S3:** nanoDSF thermogram of adsorbed TT stability timepoints at different incubation temperatures. Thermal transition temperature of TT decreased at elevated incubation temperatures.

**Table S1**: Experimental pI values for DT, TT, PT, PRN, FHA, and FIM protein antigens

| **Antigen** | **pI value** |
| --- | --- |
| Diphtheria Toxoid | 4.61-4.68, 4.64-4.69, 4.71-4.92 |
| Tetanus Toxoid | 4.88-5.07, 4.97-5.15, 5.03-5.19 |
| Pertussis Toxoid | 4.72-5.06, 5.17-5.43 |
| Filamentous Haemagglutinin | 5.54-5.71, 5.67-5.76 |
| Fimbriae 2, 3 | 4.99- 5.15, 5.37, 5.95 |
| Pertactin | 6.72-6.81, 6.99-7.07 |

**Table S2**: Protein antigen to aluminum (^27^Al ) molar ratio

| **Antigen** | **Molar ratio of protein to aluminum** |
| --- | --- |
| Diphtheria Toxoid | 4.0 x 10^-8^ |
| Tetanus Toxoid | 5.3 x 10^-9^ |
| Pertussis Toxoid | 1.5 x 10^-9^ |
| Filamentous Haemagglutinin | 7.2 x 10^-9^ |
| Fimbriae 2, 3 | 1.7 x 10^-9^ |
| Pertactin | 4.0 x 10^-9^ |

**Table S3**: Particle size distribution of pre-adsorbed (DLS) and adsorbed (LD) protein antigens

| **Antigen name** | **Hydrodynamic diameter by DLS, µm** | **Polydispersity indices from DLS measurement** | **Size distribution by LD, µm** | | |
| --- | --- | --- | --- | --- | --- |
|  |  |  | **Dv10** | **Dv50** | **Dv90** |
| PRN | 0.008 | 0.1 - 1.2 | 2.6 | 11 | 30 |
| DT | 0.07 | 0.8 - 1.1 | 2.5 | 12 | 33 |
| TT | 0.12 | 3.7 - 4.8 | 2.4 | 10 | 31 |
| PT | 0.18 | 1.4 - 2.0 | 2.9 | 10 | 32 |
| FIM | 0.078 | 1.7 - 3.0 | 2.7 | 11 | 32 |
| FHA | 0.04 | 0.4 – 0.8 | 4 | 10 | 28 |

**Table S4:** Alpha-helix and Beta-sheet content of protein antigens.

| Sample | Alpha-helix, % | Beta-sheet, % |
| --- | --- | --- |
| Pre-adsorbed PRN |  | 47 |
| AlPO_4_ Adsorbed PRN |  | 49 |
| Pre-Adsorbed DT | 47 | 37 |
| AlPO_4_ Adsorbed DT | 53 | 48 |
| Pre-Adsorbed TT | 28 | 36 |
| AlPO_4_ Adsorbed TT | 47 | 46 |
| Pre-Adsorbed FIM |  | 46 |
| AlPO_4_ Adsorbed FIM |  | 48 |
| Pre-Adsorbed FHA |  | 50 |
| AlPO_4_ Adsorbed FHA |  | 52 |

*Note: PRN, FHA, and FIM are predominantly beta-sheet proteins that also contain loops and unfolded regions.
